# The Mpox Response Among Key Populations at High Risk of or Living with HIV in Rwanda: Leveraging the Successful National HIV Control Program for More Impactful Interventions

**DOI:** 10.3390/vaccines13030307

**Published:** 2025-03-13

**Authors:** Gallican Rwibasira, Tafadzwa Dzinamarira, Jean Claude Semuto Ngabonziza, Albert Tuyishime, Ayman Ahmed, Claude Mambo Muvunyi

**Affiliations:** 1 Rwanda Biomedical Center, Kigali P.O. Box 7162, Rwanda; gallican.rwibasira@rbc.gov.rw (G.R.); claude.muvunyi@rbc.gov.rw (C.M.M.); 2 ICAP at Columbia University, Lusaka 10101, Zambia; 3 Department of Clinical Biology, University of Rwanda, Kigali 3900, Rwanda; 4 Pan-Africa One Health Institute (PAOHI), Kigali 11KG ST203, Rwanda

**Keywords:** mpox, vaccination, one health

## Abstract

Mpox, an emerging zoonotic infectious disease, presents a significant public health threat, especially among high-risk groups like female sex workers and men who have sex with men. This commentary reviews and summarizes Rwanda’s response to mpox, focusing on its intersection with HIV. Rwanda has adopted an integrated strategy to tackle both mpox and HIV by leveraging lessons and experience from the country’s success in the management of HIV and COVID-19, enhancing community engagement and health outcomes. To ensure long-term resilience, Rwanda must continue to invest in surveillance and research, expand vaccination efforts, address stigma, and foster regional impactful partnerships. Investing in fostering scientific and operational research will generate invaluable evidence that could lead to the implementation of evidence-based policymaking and cost-effective interventions.

## 1. Introduction

Mpox, formerly known as monkeypox, is a zoonotic disease caused by the monkeypox virus (MPXV), a member of the *Orthopoxvirus* genus [1]. The disease was first identified in 1958 in monkeys used for research. Human cases were first documented in 1970 in the Democratic Republic of Congo (DRC) during efforts to eradicate smallpox, an infectious disease caused by variola virus, a closely related virus [2]. Mpox outbreaks in humans were initially sporadic and largely confined to rural areas of Central and West Africa, where it became recognized as an endemic disease. The virus primarily affects individuals who come into contact with wildlife, including non-human primates, rodents, and other species that serve as natural reservoirs for the virus [3]. Although mpox has remained endemic to these regions, international outbreaks have occurred, with a significant surge in cases observed in 2022 [4].

In 2022, the World Health Organization (WHO) declared mpox a Public Health Emergency of International Concern (PHEIC) due to the rapid increase in cases outside endemic regions and the development of disease outbreaks in over 100 countries, including countries in Europe and North America [5,6,7]. The outbreak in 2022 was notable for being mainly driven by human-to-human transmission, particularly among men who have sex with men (MSM), resulting in a global concerns about sexual transmission [8]. According to the United States Centers for Disease Control and Prevention (CDC), while mpox is usually transmitted through close, sustained physical contact, the 2022–2023 global outbreak has been almost exclusively associated with sexual contact.

Certain groups are disproportionately affected by mpox, particularly MSM, female sex workers (FSWs), and individuals with compromised immune systems, including people living with HIV (PLHIV). In the context of HIV prevention and control, these populations are referred to as key populations (KPs), because they are at particularly high risk of HIV. Several factors increase the vulnerability of KPs to mpox, including social stigma, high rates of sexual contact, limited access to healthcare services, and high probability of unsafe sex practices [6].

From this perspective, we draw on existing research and expert opinions to proffer recommendations for leveraging the successful HIV control program in Rwanda to improve the mpox response in the country. To guide our perspective, we draw on the epidemiology of mpox in Rwanda, as well as our knowledge of the Rwandan health and disease surveillance system and its use in the HIV response, to highlight practical recommendations regarding challenges to the effective prevention and control of mpox in the country.

## 2. The High Risk of Mpox Among Key Populations

Several risk factors including urbanization, human and animal population movements, and changes in climate and land use are deriving the rapid increase in the prevalence and burden of infectious diseases. These factors contribute to the emergence of health issues such as mpox, Marburg virus, Rift Valley fever, and antimicrobial resistance (AMR) [5,9,10,11]. They also increase the contact between human and animal populations, which, in turn, promotes the transmission of AMR and zoonotic pathogens, including viruses [12,13,14]. Furthermore, additional sociocultural and economic risk factors attributed to human behavior and practices, such as having unprotected sex with multiple partners, are increasing KPs’ risk of acquiring sexually transmitted infections (STIs) including HIV and mpox.

## 3. Epidemiology of Mpox in Rwanda

Mpox emerged in the country for the first time in 2024, with the index case confirmed in July [15]. By 24 November 2024, Rwanda had confirmed 52 cases of mpox out of 4512 cumulative suspected cases [15,16]. Rwanda utilized robust contact tracing efforts to control the spread of the virus. Notably, nearly 50% of these cases were traced to international travelers from countries with ongoing mpox outbreaks, including DRC, where over 47,000 cases of mpox were reported [5,17]. The rest were non-traveler cases who reported sexual encounters with at least one infected traveler. This indicates the locally high risk of mpox among sex workers, including FSWs, and MSM in the country. The country, therefore, is aiming to vaccinate these KPs that are at high risk of mpox [18]. The size of the FSW and MSM populations are currently estimated to be over 98,000 and 18,000, respectively [19,20]. However, the high cost and global shortage of mpox vaccines due to the sudden resurgence in the need for vaccination driven by the global outbreak are challenging the ability of this country, with its limited resources, to carry out such a plan [21,22]. Nevertheless, Rwanda received about 5420 doses of the MVA-BN^®^ mpox vaccine through partnerships with organizations such as the European Commission, the Africa Centres for Disease Control and Prevention (Africa CDC), and the Global Alliance for Vaccines and Immunization (GAVI) [23]. These doses were delivered in the first week of October, and the vaccination campaign began immediately thereafter. The vaccination strategy is to prioritize key populations at the highest risk of exposure, using a risk-based algorithm to determine who should receive the vaccine first. This algorithm takes into account factors such as the level of risk, exposure history, and access to healthcare. Due to the limited number of vaccine doses available, the health leadership in Rwanda is initially focusing on vaccinating as many people as possible with a single dose. This strategy aims to provide broader coverage, with a plan to follow up with a second dose if resources allow and based on evolving supply and demand.

Given the high stigma and discrimination against both mpox and the sex worker community, Rwanda anticipates challenges with vaccine hesitancy and social acceptability, as seen in other countries [24,25,26]. To address these concerns, the country is leveraging existing systems from its national programs for HIV control and immunization, such as working with partners to collaboratively implement measures to enhance community engagement, health education, and promotion to counteract such potential resistance.

## 4. The Structure of the Health System in Rwanda

**Figure 1:**
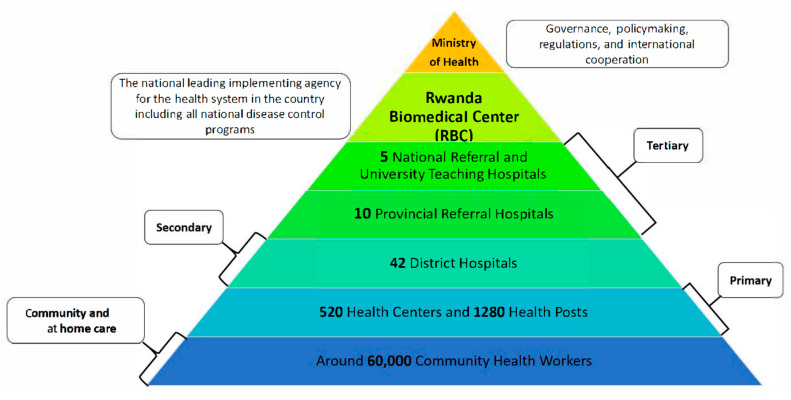
The structure of Rwanda’s health system.

Rwanda has developed a health system that aims to improve community accessibility and inclusiveness by offering multiple layers of healthcare services tailored to individuals’ needs and the complexity of their health issues (Figure 1). In addition to the 5 National Referral and University Teaching Hospitals, which, together with 10 provincial hospitals, provide tertiary healthcare services across the country, there are over 40 district hospitals offering secondary healthcare. These district hospitals are strategically distributed across Rwanda’s 30 districts, with more densely populated areas hosting multiple hospitals to better serve the population (Figure 1). Supporting these services are over 500 health centers and about 1300 health posts that provide primary healthcare services at the smallest administrative units, including cells and villages throughout the country [27,28]. One of the notable aspects of Rwanda’s health system is the Community Health Worker (CHW) program, which was established in 1995 as part of the country’s national strategy for recovery and rehabilitation following the 1994 genocide. The program was designed to address the significant loss of healthcare and public health personnel during this period. Currently, Rwanda has trained and built up a team of around 60,000 Community Health Workers who play a critical role in delivering quality healthcare and public health services at the community and household levels. CHWs are involved in the implementation of National Disease Control Programs, providing screening, testing, referrals, and treatment across more than 15 healthcare services [29,30,31]. Moreover, all these healthcare services are integrated under the National Health Insurance program that covers 92% of Rwandans. This program is independently run by the Rwanda Social Security Board (RSSB) [32,33]. This enhances the quality, reach, and coverage of healthcare services in the country, aiding Rwanda in achieving Universal Healthcare Coverage (UHC). While they are considered healthcare workers in Rwanda, CHWs are not typically classified as full “healthcare professionals” due to their volunteer status and limited medical training, though they play a crucial role in delivering primary healthcare services at the community level, particularly in rural areas; they are primarily responsible for health education, basic screening, and referring patients to healthcare facilities when necessary.

## 5. Disease Surveillance Systems in Rwanda

The country is implementing collaborative surveillance in a way that is recommended by the World Health Organization, through the cost-effective integration of various surveillance systems altogether under a joint operation unit [34]. The current structure of the collaborative surveillance system in Rwanda includes passive surveillance that is implemented by healthcare providers at healthcare facilities, syndromic surveillance that is implemented by community health workers at the community and household levels, animal and environmental surveillance systems, and wastewater epidemiology surveillance. These surveillance systems are supported by a powerful genomics analysis platform that further investigates and characterizes undetected and detected pathogens.

Despite the comprehensive and well-integrated health system in Rwanda, several challenges and limitations may impact the effectiveness of the country’s response to mpox, particularly among key populations at high risk of or living with HIV. One key challenge is limited awareness and education, as there may be insufficient public knowledge of mpox, especially among high-risk groups such as MSM, FSWs, and PLHIV, which could hinder early detection and timely intervention. The stigma surrounding sexual health and HIV-related issues further exacerbates this problem, often deterring individuals from seeking care, particularly in rural or less accessible regions. This social stigma can reduce the uptake of mpox prevention measures or delay treatment, further complicating efforts to control the spread of the disease. Additionally, resource constraints, including limited access to specialized diagnostics, vaccines, and antivirals, particularly in rural areas, may impede an effective outbreak response. While the CHW program plays a critical role in healthcare delivery, there are challenges related to the capacity and training of CHWs to manage new diseases like mpox, highlighting a need for further education and resources. Finally, although Rwanda has a robust disease surveillance system, challenges such as underreporting or delayed reporting, especially in remote regions, may hinder the timely detection of mpox cases.

## 6. Leveraging the Successful National HIV Experience for the Mpox Response in Rwanda

Rwanda is renowned for its leadership in global and public health in sub-Saharan Africa. This is indicated by the substantial progress that the country has made regarding the prevention and control of HIV, which provides valuable lessons for managing other infectious diseases such as mpox [35]. The Rwanda National HIV/AIDS Control Program (NACP) has developed and implemented an early detection and surveillance system that allows for the prompt identification of emerging diseases. Following the global alert for mpox, Rwanda quickly activated its disease surveillance units, focusing on screening and testing individuals with suspected clinical presentation, an epidemiological link to confirmed case(s), and/or a history of travel to affected areas [36].

As part of the broader public health strategy in Rwanda, high-risk groups, such as MSM and FSWs, are routinely supported to prevent and treat infection with high-risk diseases such as HIV, hepatitis C, and other STIs. This targeted surveillance system has been adapted to incorporate MPXV testing, particularly among KPs, to improve the early detection among high-risk groups [37]. The involvement of community health workers and peer educators in the provision of care to these populations has been instrumental in increasing early detection and case management that is timely and effective. Furthermore, community engagement is a cornerstone of Rwanda’s public health approach, particularly in combating infectious diseases such as HIV, malaria, tuberculosis (TB), and mpox. Public awareness campaigns have been tailored to high-risk populations to ensure that these groups are informed about the transmission, risk factors, and symptoms of mpox and how to reduce their risk of exposure. For example, MSM and FSWs have been engaged through community outreach initiatives, peer educators, and social media platforms [37]. To combat the spread of misinformation, Rwanda Biomedical Centre (RBC) has been distributing educational materials in schools, marketplaces, and public areas [38]. These materials, in both English and the local language, Kinyarwanda, have reached over 5700 schools in the form of posters. Additionally, teachers have been trained to diligently conduct daily engagement with their students to identify and clarify any misinformation.

**Figure 2:**
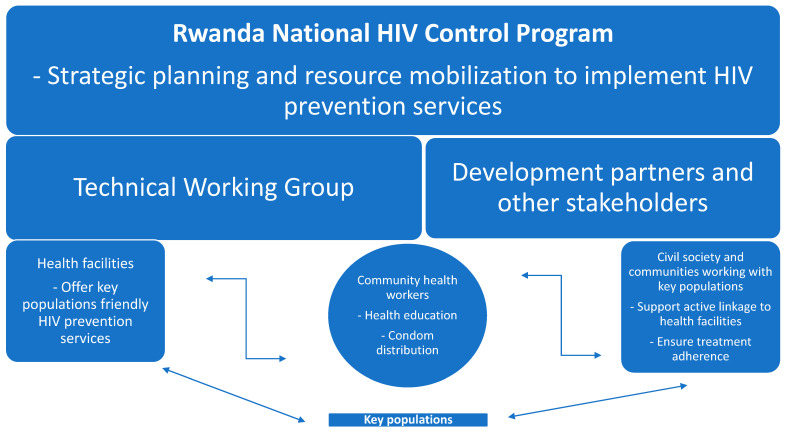
An illustration of the structure and operation of the Rwanda National HIV Control Program.

The integration of HIV and mpox education has proven effective, as many high-risk individuals already have a basic understanding of disease prevention through their participation in HIV awareness programs. Leveraging existing community networks has allowed public health authorities to disseminate information rapidly, encourage testing, and reduce stigma [38]. The stigma associated with mpox, particularly among MSM, is similar to the early stigma surrounding HIV. Rwanda’s response has emphasized the need for gender- and identity-sensitive and non-judgmental communication strategies to foster trust within these communities. Therefore, people of various gender identities and sexual orientations among the key populations are empowered to take ownership and actively contribute to the development of the communication materials and their dissemination among their peers and community, creating a safe atmosphere and an inclusive environment that enhance the uptake of information and adherence to guidelines (Figure 2).

Vaccination is a key component of mpox control, especially among high-risk populations. Rwanda’s experience with mass vaccination campaigns, such as those for COVID-19 and childhood diseases, has provided a strong foundation for its mpox vaccination efforts. Rwanda initiated a vaccination campaign on 17 September 2024 focusing on healthcare providers, community health workers, cross-border traders, hospitality employees, and other individuals at high risk [36]. In addition to vaccine distribution, promoting vaccine literacy has been crucial. To reduce the impact of misinformation and the associated vaccine hesitancy that was observed during the COVID-19 pandemic in different countries, open engagement with the populations at risk was needed [39,40]. Again, community health workers and peer educators were proven to be invaluable in improving vaccine literacy, particularly among KPs.

Rwanda’s success in managing HIV and the COVID-19 pandemic have provided a valuable framework for responding to the mpox outbreak. Key strategies from the HIV response, such as community-based healthcare, peer education, and comprehensive surveillance, have been adapted to address the mpox outbreak. The country’s HIV programs have a strong focus on reaching KPs, which has facilitated strong surveillance and the rapid identification of mpox cases within these communities.

One of the most significant lessons learned from the HIV response is the importance of integrating disease management into routine care. In Rwanda, PLHIV regularly access healthcare services, including ART. Health workers have used these regular interactions as opportunities to educate the community about mpox, provide vaccinations, and conduct mpox testing [37]. This integrated approach is anticipated to reduce the burden on healthcare systems by streamlining service delivery and optimizing resource allocation.

## 7. Recommendations

Continued efforts to enhance surveillance, early detection, community engagement, healthcare access, and vaccination, as well as efforts to strengthen the overall health system, are essential for protecting vulnerable populations and preventing future outbreaks [41]. To effectively prevent and control mpox, a comprehensive approach is necessary. While the majority of cases in Rwanda have been linked to sexual transmission, particularly among MSM, the zoonotic nature of the virus underscores the importance of strengthening global health security through a multisectoral, One Health strategy. This involves collaboration between human and animal health sectors to address the interconnectedness of these systems [9,41]. In Rwanda, mpox testing is primarily conducted through PCR testing of clinical specimens. The financing of these efforts is supported by a combination of domestic resources and international partnerships. Rwanda can continue to tailor interventions to effectively mitigate the risk of future outbreaks by understanding the specific epidemiological context in Rwanda, including the role of potential animal reservoirs and the primary modes of transmission. This is essential, especially in Rwanda, a country that is at high risk of climate change and globalization impacts that could increase outbreaks of emerging zoonotic diseases [5,10,28,42,43].

### 7.1. Strengthening Surveillance and Early Detection

Rwanda’s surveillance system for mpox is robust but could be further strengthened by increasing community-level surveillance, particularly in rural and remote areas. Given the risk of zoonotic transmission from animals to humans, especially in regions with close human–animal contact, expanding surveillance networks to implement a transdisciplinary integrated One Health surveillance and response system that incorporates human, animal (domestic and wildlife), and environmental health would enhance early detection [5,9]. Monitoring the dynamics of MPXV and other viral zoonotic diseases among potential animal hosts will provide an early warning system for new outbreaks [44]. This could involve collecting animal and environmental swab samples from within the homes of confirmed human mpox patients and testing for MPXV to better understand the epidemiology of mpox in the country. Additionally, leveraging digital tools like VisualDx could aid healthcare workers in identifying at-risk individuals and improving vaccine delivery targeting.

### 7.2. Expanding Access to Mpox Vaccination

Rwanda’s vaccination strategy is promising; however, access to vaccines remains inadequate due to limitations in their availability, access, and uptake. Increasing vaccine supply through collaboration with international partners, such as the WHO and the Africa CDC, would improve access to vaccines, particularly for the high-risk groups [5,41]. Targeted vaccination campaigns should also focus on individuals living with HIV who are at increased risk of severe mpox outcomes [5]. Rwanda could also consider implementing proactive vaccine dose-sparing strategies. These strategies, such as using fractional dosing, could extend vaccine supplies and increase the number of individuals vaccinated while offering meaningful health benefits from providing a smaller dose to a larger number of people in the high-risk population.

### 7.3. Enhancing Community Engagement to Reduce Stigma and Discrimination

Stigma remains a significant barrier in the care of high-risk populations and their uptake of healthcare services including prevention measures such as vaccines, particularly MSM. Rwanda should continue its efforts to reduce stigma through public education campaigns that emphasize the non-discriminatory nature of mpox and the importance of early detection and treatment. To enhance community engagement, healthcare authorities could organize outreach programs, where experts travel to remote and underserved areas to directly engage with local communities. Additionally, community leaders and peer educators should be actively involved, which could be done by inviting them to attend workshops or seminars with infectious disease experts, either in the capital city or through mobile platforms, where they can gain firsthand knowledge about mpox and share accurate information with their communities. Engaging community leaders and peer educators is critical for fostering trust and ensuring that high-risk individuals feel comfortable accessing healthcare services [45].

### 7.4. Promoting Cross-Border Collaboration

Rwanda has open borders and sees high amounts of cross-border movement by human and animal populations to and from countries that have reported a high mpox burden, including the global hotspot for mpox, the DRC, which presents a substantial risk. This emphasizes the need for the institutionalization of a multisectoral One Health strategy that investigates, prevents, and responds to cross-species spillover of zoonotic pathogens. Strengthening cross-border coordination with neighboring countries to enhance the implementation of International Health Regulations (IHRs 2005), including the establishment of joint surveillance systems and information-sharing platforms [46], will improve the success of regional initiatives for the prevention and control of mpox in East Africa [5,47]. This collaboration could also extend to HIV prevention and care services, improving health outcomes for KPs who may migrate across borders. Cross-border collaboration could also include the sharing of digital tools and mobile platforms to track cases and outbreaks in real time, enhancing surveillance efforts.

### 7.5. Fostering Transdisciplinary Implementation and Health System Research

It is essential for countries affected by or at risk of mpox emergence to invest in increasing their research capacity, to generate evidence from the field about the intersection between mpox, HIV, and other emerging infectious diseases [5,41]. This will have a positive impact on preparedness, prevention, and response measures because up to date evidence from the field will inform policymaking and resource mobilization, and guide the implementation of cost-effective interventions [41]. Particularly, there are huge gaps in the knowledge in terms of the risk factors for disease emergence, the prevalence of co-infection, the implications of co-infection for clinical presentation, and the challenges hindering the robustness of diagnosis and effective case management. Furthermore, investing in the development and integration of diagnostic tools, such as VisualDx, could greatly enhance the accuracy and efficiency of mpox detection in both clinical and community settings.

### 7.6. Strengthening the Implementation of the One Health Strategy to Enhance Global Health Security

Emerging infectious diseases such mpox, Marburg virus, Cholera, Ebola, Lassa fever, Rift Valley fever, Chikungunya, dengue fever, and many others are increasingly breaking out in Africa [5,9,28,42,48,49,50,51,52,53,54,55]. This necessitates the institutionalization of the One Health strategy to strengthen Global Health Security and enhance the cost-effective planning and implementation of a Pandemic Preparedness, Prevention, and Response (PPPR) framework [41]. Significant attention should be given to the silently growing pandemic of AMR [10,13,14]. Such a framework should integrate multisectoral surveillance and response systems to improve early detection and guide the implementation of cost-effective preparedness, prevention, and control measures to safeguard human, animal, and environmental health. This will help to reduce the health, socioeconomic, ecological, and political impacts of health emergencies that heavily hit poor or underserved communities. Additionally, incorporating digital health solutions into the One Health framework, such as mobile apps for data collection and analysis, could streamline responses and improve coordination across sectors.

## 8. Conclusions

Rwanda’s response to mpox, especially among high-risk groups like MSM and FSWs, has benefited from its progress in HIV prevention and management. By leveraging the existing public health infrastructure, streamlining operations, and engaging communities, Rwanda has effectively controlled the outbreak. Going forward, the country should strengthen surveillance, expand vaccine access, reduce stigma, and foster regional collaboration. Building on this success, Rwanda can enhance its approach to prevent and control other infectious diseases.

## Data Availability

No primary data were produced for this work.
